# Managing iron supply during the infection cycle of a flea borne pathogen, *Bartonella henselae*

**DOI:** 10.3389/fcimb.2013.00060

**Published:** 2013-10-18

**Authors:** MaFeng Liu, Francis Biville

**Affiliations:** ^1^Key Laboratory of Animal Disease and Human Health of Sichuan Province, Avian Disease Research Center, Institute of Preventive Veterinary Medicine, College of Veterinary Medicine, Sichuan Agricultural University, Chengdu/Ya'an, Sichuan, China; ^2^Unité des Infections Bactériennes Invasives, Département Infection et Epidémiologie, Institut Pasteur, Paris, France

**Keywords:** *Bartonella*, heme utilization, flea transmission, heme-binding protein, *Yersinia*

## Abstract

*Bartonella* are hemotropic bacteria responsible for emerging zoonoses. Most *Bartonella* species appear to share a natural cycle that involves an arthropod transmission, followed by exploitation of a mammalian host in which they cause long-lasting intra-erythrocytic bacteremia. Persistence in erythrocytes is considered an adaptation to transmission by bloodsucking arthropod vectors and a strategy to obtain heme required for *Bartonella* growth. *Bartonella* genomes do not encode for siderophore biosynthesis or a complete iron Fe^3+^ transport system. Only genes, sharing strong homology with all components of a Fe^2+^ transport system, are present in *Bartonella* genomes. Also, *Bartonella* genomes encode for a complete heme transport system. *Bartonella* must face various environments in their hosts and vectors. In mammals, free heme and iron are rare and oxygen concentration is low. In arthropod vectors, toxic heme levels are found in the gut where oxygen concentration is high. *Bartonella* genomes encode for 3–5 heme-binding proteins. In *Bartonella henselae* heme-binding proteins were shown to be involved in heme uptake process, oxidative stress response, and survival inside endothelial cells and in the flea. In this report, we discuss the use of the heme uptake and storage system of *B. henselae* during its infection cycle. Also, we establish a comparison with the iron and heme uptake systems of *Yersinia pestis* used during its infection cycle.

## Introduction

*Bartonella* are α-proteobacteria that employ arthropods for transmission and erythrocyte parasitism as a common parasitism strategy (Dehio and Sander, 1999; Schulein et al., 2001). Currently, 26 distinct *Bartonella* species have been described (Kaiser et al., 2011). *Bartonella bacilliformis, Bartonella quintana*, and *B. henselae* are the three most important human pathogens (Dehio, 2005; Florin et al., 2008; Guptill, 2010). Humans are the reservoir host for *B. bacilliformis* and *B. quintana*, in which they cause various clinical manifestations associated with both intra-erythrocytic bacteremia and endothelial cell infection (Hill et al., 1992; Maurin and Raoult, 1996). The cat is the reservoir host for *B. henselae* (Chomel et al., 2009). *B. bacilliformis* causes Oroya fever and verruga peruana (Herrer, 1953a, b). *B. quintana* causes trench fever (Vinson et al., 1969). *B. henselae* causes cat scratch disease (CSD) and bacillary peliosis (Jones, 1993). Both *B. quintana* and *B. henselae* can cause bacillary angiomatosis usually in immunodeficient patients (Spach et al., 1995; Sander et al., 1996).

## *bartonella* and their infection cycle

Each *Bartonella* species appears to be transmitted by specific bloodsucking arthropod vectors, and is highly adapted to one or several mammalian reservoir hosts, in which it causes long-lasting intra-erythrocytic bacteremia (Schroder and Dehio, 2005). Intra-erythrocytic bacteremia caused by *Bartonella* in the host has been studied in different rodent models (*B. birtlesii*/mouse, *B. tribocorum*/rat) (Boulouis et al., 2001; Schulein et al., 2001; Marignac et al., 2010). After intravenous inoculation with *in*-*vitro*-grown *B. tribocorum*, the bacteria rapidly disappeared in the circulating blood system within a few hours, and blood remained sterile for 3–4 days (Schulein et al., 2001). Endothelial cells (Dehio, 2005) or CD34^+^ progenitor cells (Mandle et al., 2005) were proposed as the primary niche. About 4–5 days post-infection, bacteria seeded from the primary niche to the bloodstream are able to invade mature erythrocytes (Schulein et al., 2001). Inside erythrocytes, bacteria multiply until reaching a steady number (eight bacteria per infected erythrocyte for *Bartonella tribocorum* in rat erythrocytes), which is maintained for the remaining lifespan of the infected cell (Schulein et al., 2001). Intra-erythrocytic bacteremia in the *B. tribocorum*-rat model is persistent for 8–10 weeks (Schulein et al., 2001). During this period, bloodsucking arthropods mediate the transmission to other susceptible hosts. This infectious procedure was also observed in a mouse model for *B. grahamii* and *B. birtlesii* (Koesling et al., 2001; Marignac et al., 2010).

## Iron/heme uptake in *bartonella*

Analysis of *Bartonella* genomes indicated that they neither encode for a siderophore biosynthesis pathway, nor for a complete Fe^3+^ transport system. *Bartonella* genomes encode for homologs of YfeABCD from *Yersinia pestis* (Perry et al., 2007) and SitABCD from avian pathogenic *E. coli*, both characterized as Fe^2+^ and Mn^2+^ inner membrane transporters (Sabri et al., 2006; Anjem et al., 2009). *Bartonella* genomes encode for a complete heme uptake system. This heme uptake system is comprised of HutA, an outer membrane heme transporter, HutB, HutC, and HmuV the three components of an inner membrane ABC transporter and a cytoplasmic heme degrading enzyme (HemS) (Parrow et al., 2009; Liu et al., 2012a). HutA from *B. quintana* contains the FRAP and NPNL domains conserved in heme transporters like HemR of *Yersinia enterocolitica* and HumR of *Yersinia pestis* (Parrow et al., 2009). Also, it was shown that HutA from *B. quintana* can apparently transport heme when expressed in *E. coli hemA* mutant strain (Parrow et al., 2009). This activity is TonB-dependent (Parrow et al., 2009). *B. tribocorum* and *B. birtlessii hutA* mutants are unable to establish bacteremia in their reservoir hosts. This result suggests that HutA is required for *Bartonella* heme uptake in mammals (Saenz et al., 2007; Vayssier-Taussat et al., 2010). After its transport into the cytoplasm, heme must be degraded to release iron. *Ex vivo*, HemS of *B. henselae* promotes the release of iron from heme when expressed in *E. coli* (Liu et al., 2012a). *In vitro*, HemS of *B. henselae* binds heme and degrades it in the presence of suitable electron donors, such as ascorbate or NADPH-cytochrome P450 reductase (Liu et al., 2012a). Interestingly, HemS activity was shown to be involved in the oxidative stress response of *B. henselae* (Liu et al., 2012a). All these above data corroborate previous *ex vivo* results demonstrating that *Bartonella* can use heme as an iron source (Sander et al., 2000). In *B. quintana*, expression of the *hutA*, *hems*, *hutB*, *hutC*, and *hmuV* is repressed by heme in an Irr-dependent manner (Parrow et al., 2009). Over expression of *fur* in the presence of heme repress *tonB* expression (Parrow et al., 2009).

## Heme-binding protein in *bartonella*

*Bartonella* express 3–5 outer membrane heme-binding proteins (Battisti et al., 2006; Minnick and Battisti, 2009). Heme-binding proteins of *Bartonella* are a group of 30–40 kDa porin-like outer membrane proteins that lack similarity with known heme receptors (Minnick et al., 2003). HbpA of *B. quintana* was shown to bind heme *in vitro* (Carroll et al., 2000). However, it did not confer a heme-binding phenotype when expressed in *E. coli* (Carroll et al., 2000). Zimmermann et al., identified a prominent heme-binding protein Pap31 (HbpA), through a heme-binding blot performed with membrane proteins from *B. henselae*. They showed that expressing Pap31 in an *E. coli K12 hemA* mutant strain restored its growth when heme was added at 30 μ M and above (Zimmermann et al., 2003). The activity of HbpA as a heme transporter was questioned by other authors. Recently, HbpA of *B. quintana* was shown to be unable to complement the *E. coli hemA* mutant in the presence of heme (Parrow, 2010). Complementation assays using the *E. coli hemA* mutant strain on solid medium in the presence of different heme concentrations also showed that HbpA of *B. birtlessii* cannot transport heme (Biville, unpublished results).

The heme uptake activity of four Hbps of *B. henselae*, expressed in an *E. coli* model strain, was investigated. All Hbps of *B. henselae* can bind heme *in vitro*. No heme transport activity was associated with expression of Hbps in *E. coli* (Liu et al., 2012b). In contrast, Hbps increase heme uptake efficiency when co-expressed with a heterologous heme transporter in an *E. coli* model strain (Liu et al., 2012b). Binding of heme by Hbps was proposed to increase its concentration around the bacteria and, as a consequence, facilitate its uptake. Another potential role for heme binding by Hbps was to provide an antioxidant barrier via heme's intrinsic peroxidase activity (Minnick and Battisti, 2009). Decreasing each Hbp amount using knockdown increases *B. henselae* sensitivity to hydrogen peroxide (Liu et al., 2012b). This antioxidant role of heme-binding proteins was evidenced to play an important role for survival of *B. henselae* in human endothelial cells and in the flea *Ctenocephalides felis*, where reactive oxygen species are produced. The expression levels of genes encoding for heme-binding proteins vary with oxygen, temperature and heme concentration (Battisti et al., 2006). One regulator, Irr, was shown to bind an “H-box” located in the promoter region of *hbp* genes (Battisti et al., 2007). Also, over-expression of RirA increased expression level of *hbpA*, *hbpD*, and *hbpE* (Battisti et al., 2007). Based on their regulatory pattern in *B. quintana*, *hbp* genes were divided into two groups. The first contained *hbpB* and *hbpC*, over expressed under conditions that mimic the gut arthropod environment [high heme concentration and low temperature (30°C), high O_2_ concentration] (Battisti et al., 2006). The transcription of *hbpA, hbpD*, and *hbpE* was increased at low heme concentrations at 37°C. HbpA, HbpD, and HbpE were suggested as being required when the free heme concentration is low, such as in blood circulation in the mammalian host. Transcription of *B. henselae hbpA* is also significantly increased at 28°C, suggesting that HbpA could protect *B. henselae* from heme toxicity in the arthropod gut (Roden et al., 2012).

## Iron sources and oxidative stress encountered during *bartonella* infection cycle

Inside the arthropod gut, *Bartonella* encounter high heme and oxygen levels. Such conditions can generate a massive oxidative stress. Thus, inside the arthropod gut, bacteria confront oxidative stress after a blood meal (Graca-Souza et al., 2006). Inside mammals, getting iron required for bacterial growth is a challenge since 99.9% of total body iron is sequestered inside the cells (Wandersman and Stojiljkovic, 2000). Outside the cells, iron is bound to transferrin in the serum or to lactoferrin in mucosal secretions (Cassat and Skaar, 2013). Another iron source in mammals is heme that is mainly contained in hemoproteins like hemoglobin. After erythrocyte lysis, most hemoglobin is bound by haptoglobin. Hemoglobin degradation allows the release of heme that is sequestered by hemopexin to prevent its toxicity (Wandersman and Stojiljkovic, 2000). Thus, obtaining iron from mammals requires transport systems allowing uptake of heme or iron bound to proteins. After inoculation in mammals, in the primary niche proposed to be endothelial cells, the intracellular iron source is iron Fe^2+^. When bacteria reach the blood stream the iron sources encountered are iron loaded transferrin, heme loaded hemopexin and hemoglobin bound to haptoglobin. Free heme is present at a low concentration (0.5 μM). *Bartonella* does not encode for a complete iron uptake system and cannot transport heme bound to hemopexin. Thus, the bacteria must use heme, stored on their surface, as an iron source. Inside erythrocytes the hemoglobin concentration is high and heme can be stored in heme-binding proteins. During their life cycle, *Bartonella* encounter heme rich environments and heme poor environments. To face this alternation, heme-binding proteins are hypothesized to play a crucial role in storing heme in the heme rich environment, and delivering it in iron/heme poor environment. The complex regulatory expression pathway of heme-binding proteins supports this hypothesis. *Bartonella* express one heme transport system HutA and control heme entry and toxicity using heme storage proteins, differentially expressed according to the bacterial infection cycle. The *Bartonella* heme uptake and storage pathways are regulated by Irr, RirA, and Fur similar to α-proteobacteria (Johnston et al., 2007). This iron/heme uptake pathway contrasts with that of another flea-borne pathogen *Yersinia pestis*.

## Iron/heme uptake and heme-binding proteins in *yersinia pestis*

**Figure 1:**
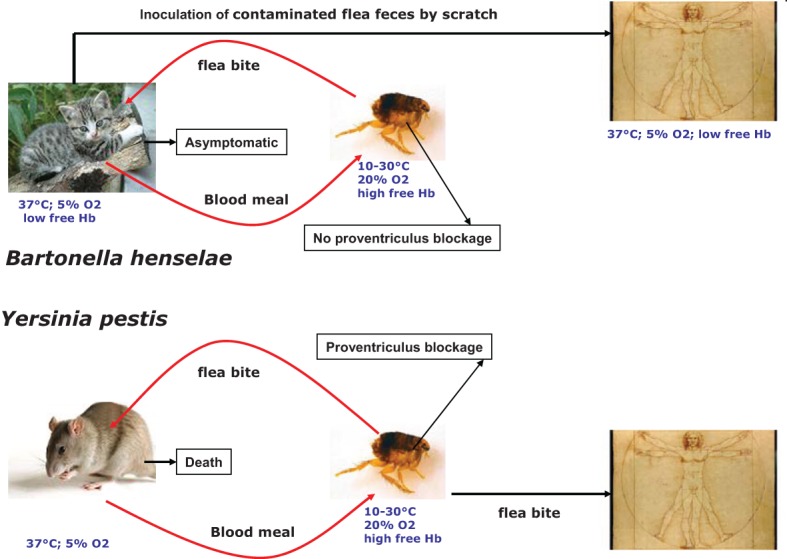
**Comparison of *Bartonella henselae* and *Yersinia pestis* transmission modes**.

**Figure 2:**
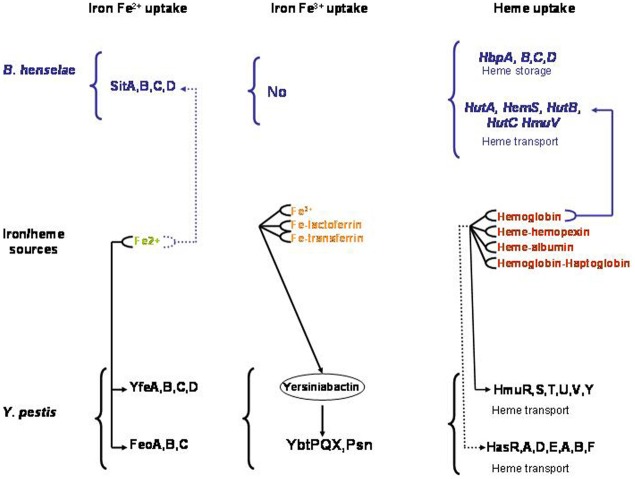
**Characterized (—) and predicted (—) Iron and heme uptake systems in *Bartonella henselae* and *Yersinia pestis***.

*Yersinia pestis*, the causative agent of plague, is transmitted mainly by infected flea bites (Chouikha and Hinnebusch, 2012) (Figure 1). Like many highly-virulent pathogenic bacteria, *Y. pestis* possess siderophore-mediated iron acquisition systems. Yersiniabactin (Ybt), the siderophore synthesized and secreted by *Y. pestis*, can remove iron from various host iron-binding proteins, such as transferrin and lactoferrin (Fetherston et al., 2010). Fe-Ybt assimilation occurs through the TonB-dependent outer membrane receptor Psn (Lucier et al., 1996). After passage through the outer membrane, Ybt-bound iron is transported into the bacterial cell by the YbtP-YbtQ ABC transporter (Fetherston et al., 1999). In *Y. pestis*, iron Fe^2+^ is transported by the YfeABCD and FeoABC systems (Perry et al., 2007). Also, *Y. pestis* encode for two heme transport systems. The *hmu* locus (hmuRSTUV) allows *Y. pestis* to use heme as well as host hemoproteins including hemoglobin, myoglobin, heme-albumin, heme-hemopexin, and hemoglobin-haptoglobin as an iron source (Hornung et al., 1996) (Figure 2). The second heme-protein acquisition system consisted of a heme receptor HasR, HasA hemophore, and HasA-dedicated ABC transporter factor HasDE, as well as a TonB homolog, HasB. However, the Has system appears not to allow the bacteria to use hemoglobin as an iron source under laboratory conditions (Rossi et al., 2001). In *Y. pestis*, the iron and heme uptake systems are Fur regulated (Gao et al., 2008). *Y. pestis*'s main transmission mode depends on the flea foregut blockage (Hinnebusch et al., 1996; Chouikha and Hinnebusch, 2012) resulting in continuous attempts to feed (Figure 1). Thus, the contaminated blood is regurgitated back into the mammalian host, where the bacteria rapidly establish an infection (Hinnebusch et al., 1996). Blockage of the flea foregut requires the activity of *hmsHFRS* gene products which synthesize and export the polysaccharide extracellular matrix required for formation of biofilm (Bobrov et al., 2008). Biofilm formation at the surface of proventricular spines is required for infection of the proventriculus (Jarrett et al., 2004). The Hms system is responsible for absorption of exogenous heme or Congo red when the bacteria are grown at 26°C (Burrows and Jackson, 1956; Surgalla and Beesley, 1969). However, the Hms system never encodes for a heme uptake system. Moreover, the stockpiled heme is not used under iron-deficient conditions (Lillard et al., 1999). Consistently, a deletion of the Hms system has no effect on *Y. pestis* virulence in mice infected by a subcutaneous route. This indicated that the Hms system is not required for the virulence of bubonic plague in a mouse model (Lillard et al., 1999). The Hms heme storage system is essential for establishing an infection within the flea midgut and to block the proventriculus (Hinnebusch et al., 1996). Heme absorbed by the Hms system protects *Y. pestis* from the nitric oxide and superoxide anion toxicity, but not from the H_2_O_2_ killing effect (Lillard et al., 1999). HmsH and HmsF were characterized as outer membrane proteins (Pendrak and Perry, 1991) while HmsR, HmsS, and HmsT are inner membrane proteins (Perry et al., 2004). HmsF and HmsR possess domains found in polysaccharide-modifying enzymes and glycosyltransferases, respectively, and HmsT belongs to the family of GGDEF proteins (Lillard et al., 1997; Ausmees et al., 2001; Perry et al., 2004). Transcription of heme acquisition operon *hmsHFRS* and *hmsT* is not regulated by the iron status inside the cells and the availability of exogenous heme (Perry et al., 2004). The temperature-dependent regulation of HmsH, HmsF, HmsR, and HmsT amount is post transcriptional and is related to a decreased stability of these proteins at 37°C (Perry et al., 2004).

## Conclusion

The two flea-borne pathogens *B. henselae* and *Y. pestis* can invade the flea gut and mammals. As a consequence, they must adapt their physiology to face the change related to these two environments. Inside the flea gut, bacteria can use heme as an iron source and must face its toxicity. Flea bite is responsible for *Y. pestis* transmission and *B. henselae* can also be transmitted by flea feces contamination (Figure 1). This transmission mode is possible since *B. henselae* is able to survive several days within feces. Inside the flea gut, *Y. pestis* forms a biofilm in the proventriculus responsible for blocking. Blocking and biofilm formation have not been described for *B. henselae*. Inside mammals, *Y. pestis* can use different iron and heme sources (Figure 2). In contrast, the sole iron/heme source for *B. henselae*, outside the cells, is heme contained in hemoglobin (Figure 2). Inside the cells, *B. henselae* could use the reduced form of iron. For *B*. *henselae*, the ability to invade erythrocytes is thus a good opportunity to obtain heme. In *Y. pestis*, no heme transport activity was associated with HmsH, HmsF, HmsR, and HmsT. Moreover, heme absorbed by the Hms system is not used as an iron source (Lillard et al., 1999). In contrast, heme-binding proteins from *B. henselae* were shown to increase the heme uptake efficiency. Inside the flea, the presence of all heme-binding proteins is required for *B. henselae* survival. The pathogenesis of *Y. pestis* is not affected by the absence of a heme storage phenotype. In contrast, disruption of one heme-binding protein in *Bartonella* impairs bacteremia establishment. In regard to the cell invasion *B. henselae* and *Y. pestis* exhibit a similar phenotype since HpbA knockdown in *B. henselae* and Hms^−^ phenotype in *Y. pestis* decrease the ability to invade cells (Lillard et al., 1999; Liu et al., 2012b). Both in *Y. pestis* and *B. henselae* the heme storage phenotype was evidenced to be involved in protection against oxidative stress. In *B. henselae*, all heme-binding proteins were associated with protection against oxidative stress generated by exposure to hydrogen peroxide. In *Y. pestis*, Hms^−^ phenotype decreases the ability to face exposure to paraquat.

For *Bartonella*, the ability to store heme looks like it is more crucial than that for *Y. pestis*. This could explain why small *Bartonella* genomes (Alsmark et al., 2004) encode for 3–5 highly homologous heme-binding proteins whose expression level is submitted to a complex regulatory pathway (Battisti et al., 2007). Since heme storage is a very important process for *Bartonella*, this can explain why decreasing the amount of only one heme-binding protein generates some defects in spite of the presence of others heme-binding proteins. In addition to the regulatory events managing expression of the various *hbp* genes, it cannot be excluded that heme-binding proteins coordinate their activity. A precise biochemical characterization of heme-binding proteins will give fruitful information about a possible cooperative activity of these outer membrane proteins.

### Conflict of interest statement

The authors declare that the research was conducted in the absence of any commercial or financial relationships that could be construed as a potential conflict of interest.

## References

[B1] Alsmark C. M. Frank A. C. Karlberg E. O. Legault B. A. Ardell D. H. Canback B. (2004). The louse-borne human pathogen *Bartonella quintana* is a genomic derivative of the zoonotic agent *Bartonella henselae*. Proc. Natl. Acad. Sci. U.S.A. 101, 9716–9721 10.1073/pnas.0305659101 15210978 PMC470741

[B2] Anjem A. Varghese S. Imlay J. A. (2009). Manganese import is a key element of the OxyR response to hydrogen peroxide in *Escherichia coli*. Mol. Microbiol. 72, 844–858 10.1111/j.1365-2958.2009.06699.x 19400769 PMC2776087

[B3] Ausmees N. Mayer R. Weinhouse H. Volman G. Amikam D. Benziman M. (2001). Genetic data indicate that proteins containing the GGDEF domain possess diguanylate cyclase activity. FEMS Microbiol. Lett. 204, 163–167 10.1111/j.1574-6968.2001.tb10880.x 11682196

[B4] Battisti J. M. Sappington K. N. Smitherman L. S. Parrow N. L. Minnick M. F. (2006). Environmental signals generate a differential and coordinated expression of the heme receptor gene family of *Bartonella quintana*. Infect. Immun. 74, 3251–3261 10.1128/IAI.00245-06 16714552 PMC1479232

[B5] Battisti J. M. Smitherman L. S. Sappington K. N. Parrow N. L. Raghavan R. Minnick M. F. (2007). Transcriptional regulation of the heme binding protein gene family of *Bartonella quintana* is accomplished by a novel promoter element and iron response regulator. Infect. Immun. 75, 4373–4385 10.1128/IAI.00497-07 17576755 PMC1951173

[B6] Bobrov A. G. Kirillina O. Forman S. Mack D. Perry R. D. (2008). Insights into *Yersinia pestis* biofilm development: topology and co-interaction of Hms inner membrane proteins involved in exopolysaccharide production. Environ. Microbiol. 10, 1419–1432 10.1111/j.1462-2920.2007.01554.x 18279344

[B7] Boulouis H. J. Barrat F. Bermond D. Bernex F. Thibault D. Heller R. (2001). Kinetics of *Bartonella birtlesii* infection in experimentally infected mice and pathogenic effect on reproductive functions. Infect. Immun. 69, 5313–5317 10.1128/IAI.69.9.5313-5317.2001 11500400 PMC98640

[B8] Burrows T. W. Jackson S. (1956). The pigmentation of *Pasteurella pestis* on a defined medium containing haemin. Br. J. Exp. Pathol. 37, 570–576 13396141 PMC2083511

[B9] Carroll J. A. Coleman S. A. Smitherman L. S. Minnick M. F. (2000). Hemin-binding surface protein from *Bartonella quintana*. Infect. Immun. 68, 6750–6757 10.1128/IAI.68.12.6750-6757.2000 11083791 PMC97776

[B10] Cassat J. E. Skaar E. P. (2013). Iron in infection and immunity. Cell Host Microbe 13, 509–519 10.1016/j.chom.2013.04.010 23684303 PMC3676888

[B11] Chomel B. B. Boulouis H. J. Breitschwerdt E. B. Kasten R. W. Vayssier-Taussat M. Birtles R. J. (2009). Ecological fitness and strategies of adaptation of Bartonella species to their hosts and vectors. Vet. Res. 40, 29 10.1051/vetres/2009011 19284965 PMC2695021

[B12] Chouikha I. Hinnebusch B. J. (2012). Yersinia–flea interactions and the evolution of the arthropod-borne transmission route of plague. Curr. Opin. Microbiol. 15, 239–246 10.1016/j.mib.2012.02.003 22406208 PMC3386424

[B13] Dehio C. (2005). Bartonella-host-cell interactions and vascular tumour formation. Nat. Rev. Microbiol. 3, 621–631 10.1038/nrmicro1209 16064054

[B14] Dehio C. Sander A. (1999). Bartonella as emerging pathogens. Trends Microbiol. 7, 226–228 10.1016/S0966-842X(99)01523-1 10447359

[B15] Fetherston J. D. Bertolino V. J. Perry R. D. (1999). YbtP and YbtQ: two ABC transporters required for iron uptake in *Yersinia pestis*. Mol. Microbiol. 32, 289–299 10.1046/j.1365-2958.1999.01348.x 10231486

[B16] Fetherston J. D. Kirillina O. Bobrov A. G. Paulley J. T. Perry R. D. (2010). The yersiniabactin transport system is critical for the pathogenesis of bubonic and pneumonic plague. Infect. Immun. 78, 2045–2052 10.1128/IAI.01236-09 20160020 PMC2863531

[B17] Florin T. A. Zaoutis T. E. Zaoutis L. B. (2008). Beyond cat scratch disease: widening spectrum of *Bartonella henselae* infection. Pediatrics 121, e1413–e1425 10.1542/peds.2007-1897 18443019

[B18] Gao H. Zhou D. Li Y. Guo Z. Han Y. Song Y. (2008). The iron-responsive Fur regulon in *Yersinia pestis*. J. Bacteriol. 190, 3063–3075 10.1128/JB.01910-07 18281395 PMC2293247

[B19] Graca-Souza A. V. Maya-Monteiro C. Paiva-Silva G. O. Braz G. R. Paes M. C. Sorgine M. H. (2006). Adaptations against heme toxicity in blood-feeding arthropods. Insect. Biochem. Mol. Biol. 36, 322–335 10.1016/j.ibmb.2006.01.009 16551546

[B20] Guptill L. (2010). Bartonellosis. Vet. Microbiol. 140, 347–359 10.1016/j.vetmic.2009.11.011 20018462

[B21] Herrer A. (1953a). Carrion's disease. II. Presence of *Bartonella bacilliformis* in the peripheral blood of patients with the benign form. Am. J. Trop. Med. Hyg. 2, 645–649 13065632

[B22] Herrer A. (1953b). Carrion's disease. I. Studies on plants claimed to be reservoirs of *Bartonella bacilliformis*. Am. J. Trop. Med. Hyg. 2, 637–643 13065631

[B23] Hill E. M. Raji A. Valenzuela M. S. Garcia F. Hoover R. (1992). Adhesion to and invasion of cultured human cells by *Bartonella bacilliformis*. Infect. Immun. 60, 4051–4058 1398917 10.1128/iai.60.10.4051-4058.1992 PMC257435

[B24] Hinnebusch B. J. Perry R. D. Schwan T. G. (1996). Role of the *Yersinia pestis* hemin storage (hms) locus in the transmission of plague by fleas. Science 273, 367–370 10.1126/science.273.5273.367 8662526

[B25] Hornung J. M. Jones H. A. Perry R. D. (1996). The hmu locus of *Yersinia pestis* is essential for utilization of free haemin and haem-protein complexes as iron sources. Mol. Microbiol. 20, 725–739 10.1111/j.1365-2958.1996.tb02512.x 9026634

[B26] Jarrett C. O. Deak E. Isherwood K. E. Oyston P. C. Fischer E. R. Whitney A. R. (2004). Transmission of *Yersinia pestis* from an infectious biofilm in the flea vector. J. Infect. Dis. 190, 783–792 10.1086/422695 15272407

[B27] Johnston A. W. Todd J. D. Curson A. R. Lei S. Nikolaidou-Katsaridou N. Gelfand M. S. (2007). Living without Fur: the subtlety and complexity of iron-responsive gene regulation in the symbiotic bacterium Rhizobium and other alpha-proteobacteria. Biometals 20, 501–511 10.1007/s10534-007-9085-8 17310401

[B28] Jones P. D. (1993). Cat scratch disease and *Rochalimaea henselae*. Med. J. Aust. 159, 211 8336627 10.5694/j.1326-5377.1993.tb137799.x

[B29] Kaiser P. O. Riess T. O'Rourke F. Linke D. Kempf V. A. (2011). Bartonella spp: throwing light on uncommon human infections. Int. J. Med. Microbiol. 301, 7–15 10.1016/j.ijmm.2010.06.004 20833105

[B30] Koesling J. Aebischer T. Falch C. Schulein R. Dehio C. (2001). Cutting edge: antibody-mediated cessation of hemotropic infection by the intraerythrocytic mouse pathogen *Bartonella grahamii*. J. Immunol. 167, 11–14 11418625 10.4049/jimmunol.167.1.11

[B31] Lillard J. W. Jr. Bearden S. W. Fetherston J. D. Perry R. D. (1999). The haemin storage (Hms+) phenotype of *Yersinia pestis* is not essential for the pathogenesis of bubonic plague in mammals. Microbiology 145(Pt 1), 197–209 10.1099/13500872-145-1-197 10206699

[B32] Lillard J. W. Jr. Fetherston J. D. Pedersen L. Pendrak M. L. Perry R. D. (1997). Sequence and genetic analysis of the hemin storage (hms) system of *Yersinia pestis*. Gene 193, 13–21 10.1016/S0378-1119(97)00071-1 9249062

[B33] Liu M. Boulouis H. J. Biville F. (2012a). Heme degrading protein HemS is involved in oxidative stress response of *Bartonella henselae*. PLoS ONE 7:e37630 10.1371/journal.pone.0037630 22701524 PMC3365110

[B34] Liu M. Ferrandez Y. Bouhsira E. Monteil M. Franc M. Boulouis H. J. (2012b). Heme binding proteins of *Bartonella henselae* are required when undergoing oxidative stress during cell and flea invasion. PLoS ONE 7:e48408 10.1371/journal.pone.0048408 23144761 PMC3483173

[B35] Lucier T. S. Fetherston J. D. Brubaker R. R. Perry R. D. (1996). Iron uptake and iron-repressible polypeptides in *Yersinia pestis*. Infect. Immun. 64, 3023–3031 8757829 10.1128/iai.64.8.3023-3031.1996 PMC174183

[B36] Mandle T. Einsele H. Schaller M. Neumann D. Vogel W. Autenrieth I. B. (2005). Infection of human CD34+ progenitor cells with *Bartonella henselae* results in intraerythrocytic presence of *B.* henselae. Blood 106, 1215–1222 10.1182/blood-2004-12-4670 15860668

[B37] Marignac G. Barrat F. Chomel B. Vayssier-Taussat M. Gandoin C. Bouillin C. (2010). Murine model for *Bartonella birtlesii* infection: new aspects. Comp. Immunol. Microbiol. Infect. Dis. 33, 95–107 10.1016/j.cimid.2008.07.011 20097421

[B38] Maurin M. Raoult D. (1996). Bartonella (Rochalimaea) quintana infections. Clin. Microbiol. Rev. 9, 273–292 8809460 10.1128/cmr.9.3.273 PMC172893

[B39] Minnick M. F. Battisti J. M. (2009). Pestilence, persistence and pathogenicity: infection strategies of Bartonella. Future Microbiol. 4, 743–758 10.2217/fmb.09.41 19659429 PMC2754412

[B40] Minnick M. F. Sappington K. N. Smitherman L. S. Andersson S. G. Karlberg O. Carroll J. A. (2003). Five-member gene family of *Bartonella quintana*. Infect. Immun. 71, 814–821 10.1128/IAI.71.2.814-821.2003 12540561 PMC145397

[B41] Parrow N. L. Abbott J. Lockwood A. R. Battisti J. M. Minnick M. F. (2009). Function, regulation, and transcriptional organization of the hemin utilization locus of *Bartonella quintana*. Infect. Immun. 77, 307–316 10.1128/IAI.01194-08 18981245 PMC2612243

[B41a] Parrow N. L. (2010). Hemin Acquisition in Bartonella quintana. PhD Dissertation. Available online at: http://etd.lib.theses/available/etd-03102010-110543/unrestricted/Parrow_umt_0136D_10080.pdf

[B42] Pendrak M. L. Perry R. D. (1991). Characterization of a hemin-storage locus of *Yersinia pestis*. Biol. Met. 4, 41–47 10.1007/BF01135556 1649616

[B43] Perry R. D. Bobrov A. G. Kirillina O. Jones H. A. Pedersen L. Abney J. (2004). Temperature regulation of the hemin storage (Hms+) phenotype of *Yersinia pestis* is posttranscriptional. J. Bacteriol. 186, 1638–1647 10.1128/JB.186.6.1638-1647.2004 14996794 PMC355957

[B44] Perry R. D. Mier I. Jr. Fetherston J. D. (2007). Roles of the Yfe and Feo transporters of *Yersinia pestis* in iron uptake and intracellular growth. Biometals 20, 699–703 10.1007/s10534-006-9051-x 17206386

[B45] Roden J. A. Wells D. H. Chomel B. B. Kasten R. W. Koehler J. E. (2012). Hemin binding protein C is found in outer membrane vesicles and protects *Bartonella henselae* against toxic concentrations of hemin. Infect. Immun. 80, 929–942 10.1128/IAI.05769-11 22232189 PMC3294634

[B46] Rossi M. S. Fetherston J. D. Letoffe S. Carniel E. Perry R. D. Ghigo J. M. (2001). Identification and characterization of the hemophore-dependent heme acquisition system of *Yersinia pestis*. Infect. Immun. 69, 6707–6717 10.1128/IAI.69.11.6707-6717.2001 11598042 PMC100047

[B47] Sabri M. Leveille S. Dozois C. M. (2006). A SitABCD homologue from an avian pathogenic *Escherichia coli* strain mediates transport of iron and manganese and resistance to hydrogen peroxide. Microbiology 152(Pt 3), 745–758 10.1099/mic.0.28682-0 16514154

[B48] Saenz H. L. Engel P. Stoeckli M. C. Lanz C. Raddatz G. Vayssier-Taussat M. (2007). Genomic analysis of Bartonella identifies type IV secretion systems as host adaptability factors. Nat. Genet. 39, 1469–1476 10.1038/ng.2007.38 18037886

[B49] Sander A. Kaliebe T. Bredt W. (1996). [Bartonella (Rochalimaea) infections: cat-scratch disease and bacillary angiomatosis]. Dtsch. Med. Wochenschr. 121, 65–69 10.1055/s-2008-1042973 8565812

[B50] Sander A. Kretzer S. Bredt W. Oberle K. Bereswill S. (2000). Hemin-dependent growth and hemin binding of *Bartonella henselae*. FEMS Microbiol. Lett. 189, 55–59 10.1111/j.1574-6968.2000.tb09205.x 10913865

[B51] Schroder G. Dehio C. (2005). Virulence-associated type IV secretion systems of Bartonella. Trends Microbiol. 13, 336–342 10.1016/j.tim.2005.05.008 15935675

[B52] Schulein R. Seubert A. Gille C. Lanz C. Hansmann Y. Piemont Y. (2001). Invasion and persistent intracellular colonization of erythrocytes. A unique parasitic strategy of the emerging pathogen Bartonella. J. Exp. Med. 193, 1077–1086 10.1084/jem.193.9.1077 11342592 PMC2193435

[B53] Spach D. H. Kanter A. S. Dougherty M. J. Larson A. M. Coyle M. B. Brenner D. J. (1995). Bartonella (Rochalimaea) quintana bacteremia in inner-city patients with chronic alcoholism. N. Engl. J. Med. 332, 424–428 10.1056/NEJM199502163320703 7529895

[B54] Surgalla M. J. Beesley E. D. (1969). Congo red-agar plating medium for detecting pigmentation in *Pasteurella pestis*. Appl. Microbiol. 18, 834–837 5370459 10.1128/am.18.5.834-837.1969 PMC378096

[B55] Vayssier-Taussat M. Le Rhun D. Deng H. K. Biville F. Cescau S. Danchin A. (2010). The Trw type IV secretion system of Bartonella mediates host-specific adhesion to erythrocytes. PLoS Pathog. 6:e1000946 10.1371/journal.ppat.1000946 20548954 PMC2883598

[B56] Vinson J. W. Varela G. Molina-Pasquel C. (1969). Trench fever. 3. Induction of clinical disease in volunteers inoculated with *Rickettsia quintana* propagated on blood agar. Am. J. Trop. Med. Hyg. 18, 713–722 5810799

[B57] Wandersman C. Stojiljkovic I. (2000). Bacterial heme sources: the role of heme, hemoprotein receptors and hemophores. Curr. Opin. Microbiol. 3, 215–220 10.1016/S1369-5274(00)00078-3 10744995

[B58] Zimmermann R. Kempf V. A. Schiltz E. Oberle K. Sander A. (2003). Hemin binding, functional expression, and complementation analysis of Pap 31 from *Bartonella henselae*. J. Bacteriol. 185, 1739–1744 10.1128/JB.185.5.1739-1744.2003 12591895 PMC148071

